# Short-term association of particulate matter and cardiovascular disease mortality in Shanghai, China between 2003 and 2020

**DOI:** 10.3389/fpubh.2024.1388069

**Published:** 2024-04-05

**Authors:** Bo Fang, Jing Wei, Lei Chen, Shan Jin, Qi Li, Renzhi Cai, Naisi Qian, Zhen Gu, Lei Chen, Romain Santon, Chunfang Wang, Weimin Song

**Affiliations:** ^1^School of Public Health, Fudan University, Shanghai, China; ^2^Shanghai Municipal Center for Disease Control and Prevention, Shanghai, China; ^3^Department of Atmospheric and Oceanic Science, Earth System Science Interdisciplinary Center, University of Maryland, College Park, MD, United States; ^4^Vital Strategies, Shanghai, China

**Keywords:** particulate matter, air pollution, cardiovascular disease, mortality time-series study, Shanghai

## Abstract

**Objective:**

Evidence regarding the effects of particulate matter (PM) pollutants on cardiovascular disease (CVD) mortality remains limited in Shanghai, China. Our objective was to thoroughly evaluate associations between PM pollutants and CVD mortality.

**Methods:**

Daily data on CVD mortality, PM (PM_10_ and PM_2.5_) pollutants, and meteorological variables in Shanghai, China were gathered from 2003 to 2020. We utilized a time-series design with the generalized additive model to assess associations between PM pollutants and CVD mortality. Additionally, we conducted stratified analyses based on sex, age, education, and seasons using the same model.

**Results:**

We found that PM pollutants had a significant association with CVD mortality during the study period. Specifically, there was a 0.29% (95%CI: 0.14, 0.44) increase in CVD mortality for every 10 μg/m3 rise in a 2-day average (lag01) concentration of PM_10_. A 0.28% (95% CI: 0.07, 0.49) increase in CVD mortality was associated with every 10 μg/m3 rise in PM_2.5_ concentration at lag01. Overall, the estimated effects of PM_10_ and PM_2.5_ were larger in the warm period compared with the cold period. Furthermore, males and the older adult exhibited greater susceptibility to PM_10_ and PM_2.5_ exposure, and individuals with lower education levels experienced more significant effects from PM_10_ and PM_2.5_ than those with higher education levels.

**Conclusion:**

Our findings suggested that PM pollutants have a substantial impact on increasing CVD mortality in Shanghai, China. Moreover, the impacts of air pollution on health may be altered by factors such as season, sex, age, and educational levels.

## 1 Introduction

Over the last two decades, increasing studies have suggested the detrimental consequences of atmospheric air pollution on human wellbeing (1–3). Among the various air pollutants, particulate matter (PM) has been identified as the predominant pollutant (4). PM, which includes PM_10_ and PM_2.5_, is produced by combustion sources or atmospheric chemical change (5). PM pollution has become a major global public concern, especially in developing nations (6–8). According to the Global Burden of Disease Study 2019, there were ~3.5 million deaths from cardiovascular diseases (CVDs) due to PM pollutants in 2019 (9).

CVD stands out as the predominant health challenge faced by China's healthcare system (10). Over recent years, CVD has posed a substantial health burden on China's healthcare system, requiring comprehensive strategies and interventions to address its multifaceted impact on public health (11). Moreover, there is an anticipation of an increase in the prevalence and mortality of CVD in the coming decade. The swift progress of industrialization and urbanization has led to a severe decline in air quality in China over the past few decades (12). To mitigate the health and economic burdens associated with CVD, it is crucial to decrease concentrations of PM_10_ and PM_2.5_ by strengthening individual protective measures. While various studies highlight the adverse influence of air pollutants on the development or worsening of CVD (13), it is noteworthy that the impacts of PM_10_ and PM_2.5_ on CVD mortality differ in diverse geographical areas. It becomes crucial to recognize the health consequences of air pollution by relying on data specific to the local context. Hence, it is imperative to undertake a comprehensive investigation to analyze thoroughly how PM pollutants affect the wellbeing of residents in Shanghai, China.

Existing research on the modifying impact of socioeconomic status on air pollution-related health outcomes has yielded inconsistent findings (14, 15). The majority of these investigations have been carried out in developed nations, with only a limited number focusing on Asia. There remains a critical gap in studies conducted in cities of developing countries, where the characteristics of outdoor air pollution, meteorological conditions, and sociodemographic patterns may diverge from those in North America and Europe. A more comprehensive understanding of these modifying factors is crucial for informing public policy, facilitating risk assessment, and establishing standards, particularly in developing countries with a scarcity of existing studies.

Thus, our objective is to evaluate associations between PM pollutants and CVD mortality in Shanghai, China during the period from 2003 to 2020. We further conducted stratified analyses based on age, sex, education, and season to pinpoint the susceptible subgroups.

## 2 Materials and methods

### 2.1 Mortality

Shanghai, a metropolis renowned for its dense population, intertwines urban and suburban districts, alongside counties, covering an expansive area of 6, 341 km^2^. The city, positioned on the southern estuary of the Yangtze River with the Huangpu River meandering through its landscape, reflects a dynamic blend of geography. At the end of 2020, the city accommodated an impressive population of 24.9 million inhabitants. Our study concentrated on the 16 distinct districts within Shanghai, allowing us to meticulously explore the multifaceted dimensions of our research locale and derive comprehensive insights.

Daily death data were collected from the Shanghai Municipal Center for Disease Control and Prevention during the study period from 2003 to 2020. We further extracted the CVD mortality data according to International Classification Diseases, 10th revision.

### 2.2 Air pollution and meteorology data

Daily PM_10_ and PM_2.5_ concentrations in Shanghai were sourced from ChinaHighAirPollutants (CHAP) database, featuring a spatial resolution of 1 × 1 km (16, 17). This dataset has found extensive application in prior research endeavors. The meticulous alignment of observed ground data with anticipated PM_10_ and PM_2.5_ levels is underscored by robust cross-validation coefficients of determination, registering at 0.92 and 0.90, respectively. Moreover, the associated root mean square errors for PM_10_ and PM_2.5_ stood at 10.76 and 21.12 μg/m3, respectively, affirming the reliability of the collected data. To factor in meteorological influences on CVD mortality, we meticulously procured daily temperature and humidity data from Shanghai Meteorological Bureau database.

### 2.3 Statistical analysis

Separate analyses were conducted using a generalized additive model (GAM) to evaluate associations between PM and CVD mortality in this time-series study (18). Initially, we formulated foundational models for various mortality outcomes, excluding the consideration of PM pollutants. In these models, we integrated natural spline (ns) function for time and meteorological variables, providing a versatile modeling tool capable of capturing non-linear and non-monotonic associations between mortality and temporal and weather-related factors. To ascertain the suitable degrees of freedom (df) for time trend, we utilized the partial autocorrelation function (PACF). Through comprehensive evaluation of residual and PACF plots, we scrutinized residuals for identifiable patterns and autocorrelation in the main model. The main model incorporated following covariates: (1) a ns function with 7 df per year, (2) an indicator day-of-week variable to accommodate short-term weekly variations, and (3) ns functions with 6 df for temperature and 3 df for relative humidity, respectively. In the main analyses, a 2-day moving average (lag01) was applied to evaluate the current- and previous-day concentrations of PM_10_ and PM_2.5_, as this frequently yielded the most significant effect estimate in earlier investigations. The equation is as below:


$$\begin{array}{l} logE(Yt)=\beta Z_{t}+DOW+ns(time,\;6)+ns(temp,\;6)\\ \;+ns\;(humidity,\;3)+intercept \end{array}$$

where *E(Yt)* denoted the anticipated number of deaths on day *t*; β, signifying the logarithmic relative rate of CVD mortality linked with a one-unit escalation in PM pollutant concentrations; *Z*_*t*_ denoted pollutant concentrations on day *t*; *DOW* denoted a dummy variable accounting for the day of the week; *ns(time,6)* denoted the ns function of calendar time with 6 df; and *ns(temp, 6)* and *ns(rh, 3)* denoted the ns functions for temperature and relative humidity with 6 and 3 df, respectively. Current-day temperature and relative humidity were incorporated were applied in this model.

Furthermore, individual lags spanning 0, 1, 2, and 3 days were scrutinized to discern lag patterns. In the exploration of potential individual-level effect modifiers for PM_10_ and PM_2.5_, stratification analyses were implemented based on age (45–64, and ≥65), sex (male, female), and educational level (high, >9 years of education; low, ≤ 9 years of education). To identify the seasonal effect, we further conducted separate analyses for the warm period (April to September) and the cold period (October to March). Employing the same methodology as the preior study, we amalgamated exposure–response (E-R) relationship curves for PM_10_ and PM_2.5_, respectively.

Sensitivity analyses aimed at assessing the robustness of our results were performed by altering df for time (8 and 9), temperature (7 and 8), and relative humidity (4 and 5), respectively.

The analyses were conducted using R (version 3.5.1) with the “mgcv” package. The results are expressed as the percentage associated with its 95% confidence interval (CI) in CVD mortality with every 10 μg/m^3^ increase of PM_10_ and PM_2.5_.

## 3 Results

### 3.1 Descriptive results

Table 1 summarizes descriptive statistics of CVD mortality, PM pollutants, and meteorological data in Shanghai, China between 2003 and 2020. Daily average count of CVD mortality stood at 50.7, with a higher count observed in females compared to males. The mean concentrations of PM_10_ exhibited a range from 14.4 to 402.8 μg/m^3^, with a mean concentration of 77.0 μg/m^3^. The mean concentrations of PM_2.5_ varied between 8.6 and 326.2 μg/m^3^, with a mean concentration of 48.2 μg/m^3^.

**Table 1:** Summary descriptive statistics on average numbers of daily cardiovascular disease (CVD) mortality, particulate matter, and weather conditions in Shanghai, China from 2003 to 2020.

	**Mean (SD)**	**Min**	**P25**	**P50**	**P75**	**Max**
**CVD mortality**						
Male	23.4 (9.4)	3.0	16.0	22.0	29	66
Female	27.3 (10.8)	5.0	19.0	26	34	76
Total death counts	50.7 (18.9)	14	36	48	62	132
**Particulate matter (**μ**g/m**^3^**)**						
PM_10_	77.0 (35.4)	14.4	50.7	70.0	96.3	402.8
PM_2.5_	48.2 (25.2)	8.6	29.9	43.4	61.0	326.2
**Meteorological measures**						
Temperature (°C)	17.4 (8.9)	−6.1	9.6	18.4	24.7	35.7
Humidity (%)	71.6 (12.4)	23.0	64.0	72.0	81.0	100.0

CVD, cardiovascular disease; SD, standard deviation; PM_10_, particles with a diameter of 10 micrometers or less; PM_2.5_, particles with a diameter of 2.5 micrometers or less.

**Figure 1:**
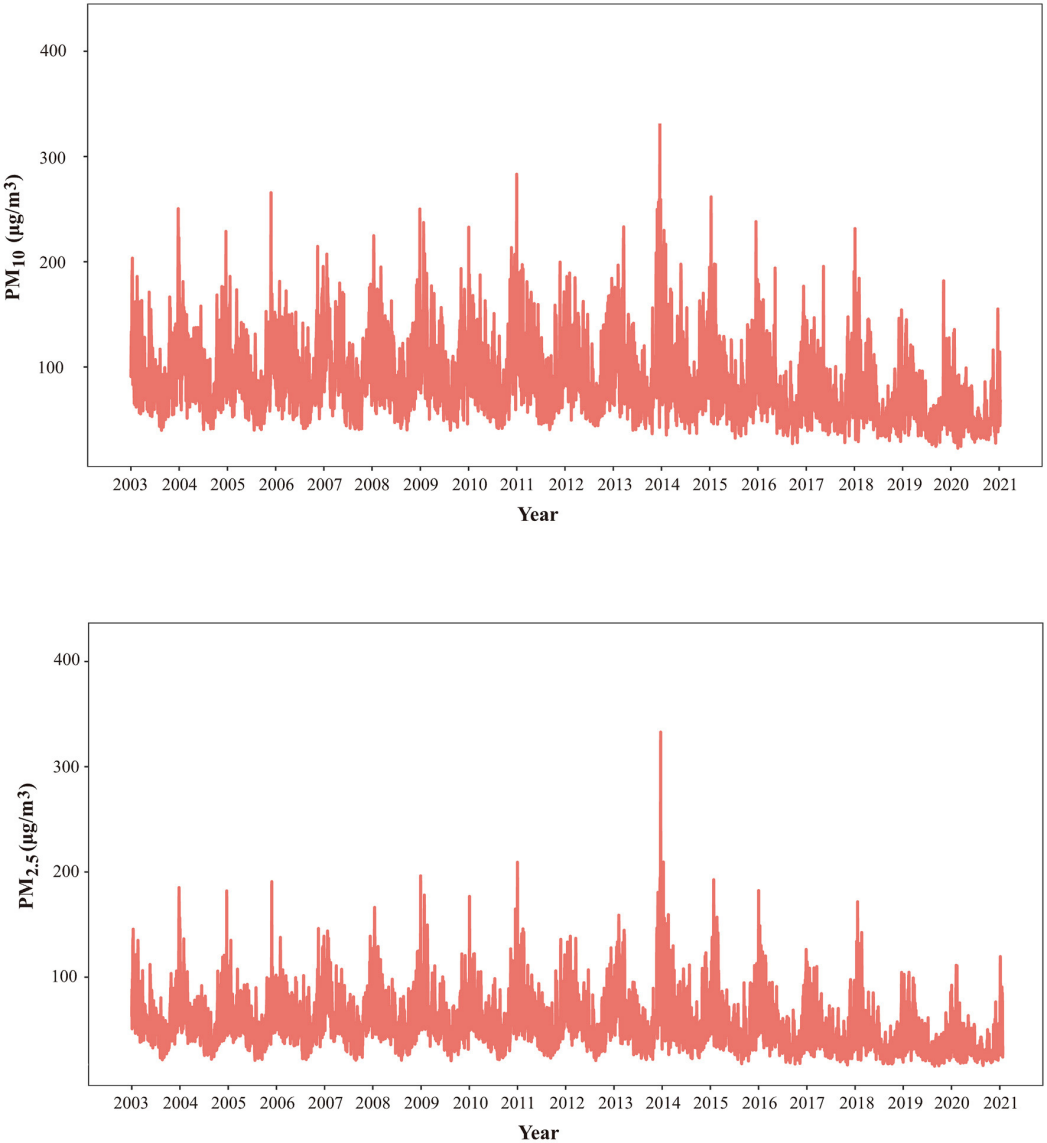
Time series of PM_10_ and PM_2.5_ in Shanghai, China from 2003 to 2020. CVD, cardiovascular disease; SD, standard deviation; PM_10_, particles with a diameter of 10 micrometers or less; PM_2.5_, particles with a diameter of 2.5 micrometers or levss.

The overall daily concentrations of PM_10_ and PM_2.5_ depicted an annual trend characterized by relative stability. Figure 1 illustrates the patterns of both pollutants, revealing consistent periodic fluctuations. It should be noted that these concentrations remained stable throughout the year, with the winter period emerging as the period of peak concentrations for both PM_10_ and PM_2.5_.

### 3.2 Regressive results

Table 2 summarizes increases in CVD mortality associated with every 10 μg/m^3^ increase of PM_10_ and PM_2.5_ from 0 to 3 lag days and lag01 days. Generally, in the whole-period analyses, PM_10_ and PM_2.5_ were significantly associated with mortality from CVD in Shanghai at different lag patterns. For the lag0 day, the results suggested that every 10 μg/m^3^ increase of PM_10_ and PM_2.5_ was significantly associated with an increase of 0.18% (95% CI: 0.04, 0.31) and 0.21% (95% CI: 0.03, 0.39) in CVD mortality, respectively. The highest estimates for the impact of PM_10_ and PM_2.5_ on CVD mortality were observed at lag01. There was a significant elevation in CVD mortality risk by 0.29% (95% CI: 0.14, 0.44) and 0.28% (95% CI: 0.07, 0.49) for every 10 μg/m^3^ increase in PM_10_ and PM_2.5_ at lag01, respectively.

**Table 2:** . Percent changes and 95% confidence intervals in cardiovascular disease associated with a 10 μg/m^3^ in PM_2.5_ and PM_10_ during different lag days in Shanghai, China from 2003 to 2020.

**Lag days**	**Cardiovascular disease mortality**
**PM**_10_	
Lag 0	0.18 (0.04, 0.31)
Lag 1	0.27 (0.13, 0.4)
Lag 2	0.21 (0.08, 0.35)
Lag 3	0.19 (0.06, 0.32)
Lag 01	0.29 (0.14, 0.44)
**PM**_2.5_	
Lag 0	0.21 (0.03, 0.39)
Lag 1	0.21 (0.02, 0.39)
Lag 2	0.13 (−0.05, 0.31)
Lag 3	0.11 (−0.07, 0.28)
Lag 01	0.28 (0.07, 0.49)

CVD, cardiovascular disease; SD, standard deviation; PM_10_, particles with a diameter of 10 micrometers or less; PM_2.5_, particles with a diameter of 2.5 micrometers or less.

**Figure 2:**
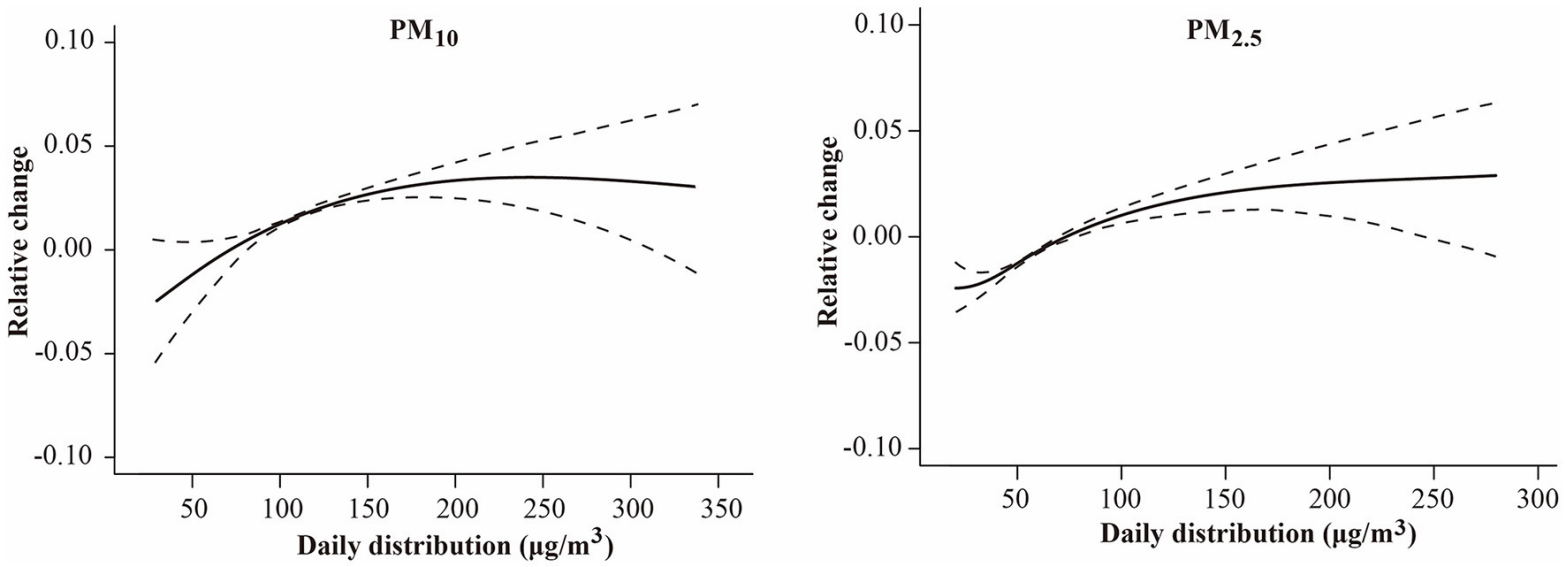
The exposure–response relationship curves between PM (PM_10_ and PM_2.5_) concentrations and daily cardiovascular disease mortality in Shanghai, China from 2003 to 2020. The *y*-axis can be interpreted as the relative change from the mean effect of PM_10_ and PM_2.5_ on mortality. The solid line represents the mean estimate, and the dashed lines represent 95% confidence intervals. CVD, cardiovascular disease; SD, standard deviation; PM_10_, particles with a diameter of 10 micrometers or less; PM_2.5_, particles with a diameter of 2.5 micrometers or less.

Figure 2 shows the E-R curves between PM_10_ and PM_2.5_ and CVD mortality. Notably, no discernible thresholds were identified, signifying positive associations persisting even within lower exposure ranges.

The estimates of PM_10_ and PM_2.5_ on people stratified by sex, age, and educational levels are summarized in Table 3. Our analysis revealed that these exerted modifying factors influenced the associations between PM_10_ and PM_2.5_ and CVD mortality. Males exhibited a higher susceptibility of males to PM_10_ and PM_2.5_ exposure compared to females. For males, there was a significant elevation in CVD mortality by 0.32% (95% CI: 0.10, 0.54) and 0.35% (95% CI: 0.05, 0.65) for every 10 μg/m^3^ increase in PM_10_ and PM_2.5_ at lag01, respectively. For females, every 10 μg/m^3^ increase in PM_10_ was associated with an elevation of 0.27% (95% CI: 0.06, 0.47) in CVD mortality, whereas PM_2.5_ had no significant association [0.21% (95% CI: −0.07, 0.49)]. Individuals with low education levels had a higher association of CVD mortality [0.32% (95% CI: 0.12, 0.51)] from PM_10_-related effects compared to those with high education levels [0.25% (95% CI: 0.01, 0.48)]. The effect estimates of PM_2.5_ were significant in the low educational attainment group [0.27% (95% CI: 0.01, 0.54)] but not in the residents with high educational levels [0.28% (95% CI: −0.05, 0.60)]. No significant association of PM_10_ and PM_2.5_ was observed in the adults aged 45–64 years. Conversely, among adults aged ≥ 65 years, the estimates for PM_10_ and PM_2.5_ were significant, approximately doubling in magnitude compared to those aged 45–64 years.

**Table 3:** Percent changes and 95% confidence intervals in cardiovascular disease mortality associated with a 10 μg/m^3^ in PM_10_ and PM_2.5_ at lag01 in Shanghai, China from 2003 to 2020, classified by sex, age, and education.

**Variables**	**PM_10_**	**PM_2.5_**
**Sex**	
Male	0.32 (0.1, 0.54)	0.35 (0.05, 0.65)
Female	0.27 (0.06, 0.47)	0.21 (−0.07, 0.49)
**Education**	
High	0.25 (0.01, 0.48)	0.28 (−0.05.0.6)
Low	0.32 (0.12, 0.51)	0.27 (0.01, 0.54)
**Ages (years)**	
45–64	0.14 (−0.38, 0.67)	0.21 (−0.55, 0.98)
≥65	0.32 (0.16, 0.48)	0.31 (0.09, 0.53)

CVD, cardiovascular disease; SD, standard deviation; PM_10_, particles with a diameter of 10 micrometers or less; PM_2.5_, particles with a diameter of 2.5 micrometers or less.

Table 4 summarizes the impact of PM_10_ and PM_2.5_ on CVD mortality stratified by seasons. Notably, every 10 μg/m^3^ increase in PM_10_ exhibited a significant association with an increase of 0.46% (95% CI: 0.12, 0.81) and 0.21% (95% CI: 0.03, 0.39) in CVD mortality during the warm and cold period, respectively. We found every 10 μg/m^3^ increase in PM_2.5_ associated with 0.44% (95% CI: −0.07, 0.95) and 0.19% (95% CI: −0.05, 0.45) in CVD mortality during the warm and cold period though there was no significant association. Moreover, the impact assessments of PM_10_ and PM_2.5_ in the warm season exhibited an ~2-fold increase compared to their counterparts in the cold season. These estimated effects displayed variability when stratified by sex, age, and education groups, emphasizing the nuanced nature of the associations across different demographic categories.

**Table 4:** Percent changes and 95% confidence intervals in cardiovascular disease (CVD) mortality associated with a 10 μg/m^3^ in PM_10_ and PM_2.5_ at lag01 in Shanghai, China from 2003 to 2020 in different seasons.

**Variable**	**PM**_**10**_		**PM**_**2.5**_	
	**Warm period**	**Cold period**	**Warm period**	**Cold period**
CVD mortality	0.46 (0.12, 0.81)	0.21 (0.03, 0.39)	0.44 (−0.07, 0.95)	0.19 (−0.05, 0.43)
**Sex**				
Male	0.52 (0.03, 1.02)	0.22 (−0.03, 0.48)	0.50 (−0.23, 1.23)	0.26 (−0.08, 0.6)
Female	0.41 (−0.05, 0.88)	0.20 (−0.04, 0.43)	0.40 (−0.28, 1.09)	0.13 (−0.19, 0.44)
**Education**				
High	0.54 (−0.002, 1.09)	0.14 (−0.13, 0.42)	0.57 (−0.22, 1.37)	0.17 (−0.2, 0.54)
Low	0.42 (−0.03, 0.86)	0.25 (0.02, 0.48)	0.37 (−0.28, 1.03)	0.20 (−0.11, 0.5)
**Ages (years)**				
45–64 years	1.22 (0.08, 2.36)	−0.19 (−0.82, 0.43)	1.48 (−0.18, 3.18)	−0.14 (−0.97, 0.7)
≥65	0.43 (0.07, 0.80)	0.25 (0.07, 0.44)	0.41 (−0.12, 0.95)	0.23 (−0.02, 0.48)

In the sensitivity analyses, the effect estimates of CVD mortality remained consistent with each 10 μg/m^3^ increment of PM_10_ and PM_2.5_, even after adjusting for temperature, humidity, and the annual time trend (specific data not presented). These findings underscore the robustness of the observed associations across various scenarios and highlight the reliability of the study results.

## 4 Discussion

The comprehensive study meticulously explored the association between PM pollutants and CVD mortality in Shanghai, China. The outcomes revealed significant positive associations, indicating an increased risk of CVD mortality associated with PM pollutants. Additionally, our findings underscored the nuanced impact of PM pollutants, emphasizing their modification by seasonal variations and individual sociodemographic factors in shaping the intricate landscape of public health outcomes.

The daily mean concentration of PM_10_ was 77.0 μg/m^3^ throughout the study period, which surpassed ambient air quality standards (AQS) set in China but remained below secondary standard (with primary standard at 50 μg/m3 and the secondary at 150 μg/m3). Concurrently, the mean PM_2.5_ concentration stood at 48.2 μg/m3, exceeding the AQS primary standard while staying beneath the secondary standard (with the primary at 35 μg/m3 and the secondary at 75 μg/m3). PM pollutants exhibited relatively stable patterns, with peak levels observed during the winter months in the current study. This phenomenon is attributed to coal burning, a pivotal source of heat and energy in China during winter (19). Furthermore, low temperatures experienced in winter can contribute to inadequate dispersion of air pollutants (20), ultimately leading to peak values during this season. Additionally, the surge in the quantity of vehicles over recent decades has significantly contributed to environmental air pollution in Shanghai, China. Vehicle emissions, consisting of various pollutants, have become a substantial source of airborne contaminants, further impacting air quality (21). This multifaceted interplay of seasonal conditions, coal burning, and vehicular emissions underscores the dynamic nature of air pollution in Shanghai, emphasizing the need for comprehensive strategies to address diverse pollution sources and mitigate their health implications.

We found a positive association between PM pollutants and CVD mortality. The impact of both PM_10_ and PM_2.5_ on CVD mortality reached its peak at lag01 days. Similarly, Kan et al. suggested that every 10 μg/m3 rise in PM_10_ was linked to a 0.27% (95% CI: 0.10, 0.44) increase in CVD mortality in Shanghai from 2001 to 2004 (18). Tong et al. similarly documented that every 10 μg/m3 increase in PM_10_ was associated with a 0.19% rise in CVD mortality in Tianjin, China (22). Research conducted in Wuhan, China revealed that each 10 μg/m3 rise in PM_10_ was associated with a 0.57% increase in CVD mortality (23). Our results were in line with the findings reported in earlier studies. Nevertheless, some studies have reported different findings. The differences in these results could be attributed to various factors, including: (1) chemical compositions of PM exhibit regional variations, potentially resulting in diverse effects on CVD mortality (24). Variability in the sources and constituents of PM in different regions can lead to distinct health outcomes; (2) differences in the age distribution of the exposed populations across various regions can introduce variations in susceptibility to PM pollutants (25), influencing the overall impact on CVD mortality; (3) the observed differences may stem from variations in the sensitivity of different regions to PM pollutants, reflecting diverse environmental and demographic contexts (26). Recognizing and understanding these nuanced factors are essential for comprehensively interpreting and addressing the complexities of PM-related health effects in distinct geographical settings.

Subgroup analysis further revealed significant associations between PM pollutants and CVD mortality across various sex, age, and education groups. In the sex-stratified analysis, males exhibited greater vulnerability to PM pollution compared to females. This observation aligns with the results reported in several preceding studies (27). The reasons behind sex-specific observations remain unclear and warrant further investigation. A reasonable hypothesis is that men and women have various physiological structures, engage in distinct social behaviors, and possess various characteristics, such as routine activities, occupation, and smoking habits. These factors could interact with air pollutants, potentially resulting in diverse health outcomes between males and females (28). Similar to prior studies (29, 30), in the age-stratified analysis, the results underscored that older individuals (≥65 years) exhibited heightened vulnerability to the impacts of PM_10_ and PM_2.5._ This susceptibility among the older adult may be attributed to diverse physiological functions and distinct personal behaviors prevalent in different age groups (31). The intricate interplay between age-related factors, such as physiological changes and unique lifestyle patterns, could contribute to the observed variations in susceptibility to PM pollutants.

The influence of sociodemographic patterns on health indicators, including mortality, has been well-established over time. In recent research, an increasing focus has been placed on investigating the impact of socioeconomic status on the susceptibility of subpopulations to the unfavorable impacts of air pollution, particularly concerning PM pollutants. In line with existing research (32, 33), we also observed a significantly higher vulnerability to PM pollutants among individuals with lower education levels. The increased risk estimates in less-educated populations could stem from environmental health disparities and inequities linked to socioeconomic status. The impact of air pollution on health is intricately linked to socioeconomic factors, particularly educational attainment. Those with lower levels of education may exhibit heightened sensitivity to health risks associated with air pollution, often attributed to a greater prevalence of preexisting conditions that elevate the mortality risk linked to exposure. Additionally, individuals with lower educational levels may face inadequate medical care for existing health issues (34). Living in disadvantaged conditions can amplify this modification effect, as limited access to nutritious foods among this demographic may result in decreased consumption of vitamins and antioxidant polyunsaturated fatty acids protecting against the adverse effects of PM exposure. Furthermore, exposure patterns may contribute to modifying effects based on education levels, with individuals of lower education being less likely to have air purifiers, more prone to residing near traffic roads, and experiencing coexposures of substandard housing or occupation. Recognizing variations in population susceptibility to PM, it is possible to more effectively adapt the creation of public health preventative initiatives to lower the burden of illnesses in Shanghai. Customized approaches, considering factors such as age, sex, and educational background, can enhance the efficacy of interventions, ensuring targeted and impactful measures to safeguard public health.

Concerning seasonal effects, our findings suggested association between PM pollutants and CVD mortality was higher in the warm season compared to the cold season. These findings align with several prior studies (35, 36). However, contradictory findings were documented in several other research (22, 37). Hence, the seasonal differences in the impact of PM pollutants on CVD mortality remain uncertain. We speculate that the differing effects of PM pollutants on CVD mortality in different seasons could be attributed to several factors: (1) warmer temperatures may exacerbate the physiological stress on the cardiovascular system (38), making individuals more susceptible to the harmful outcomes of PM pollutants; (2) seasonal variations in meteorological conditions, such as increased humidity and temperature in the warm season, can influence the dispersion and PM pollutants, intensifying their impact on health; (3) different seasonal activities may contribute to varying exposure patterns. For instance, in the warmer weather, people may spend more time outside, which would expose them to increased outdoor air pollution; (4) biological interactions between PM pollutants and other seasonal factors, such as allergens or infectious agents, may amplify the health effects during warmer seasons; and (5) seasonal variations in population behavior, like increased travel or outdoor events during warmer months, can contribute to elevated exposure levels. Understanding these reasons can contribute to more targeted interventions and policies aimed at reducing the health impact of PM pollutants, especially during seasons when the effects are more pronounced.

Several limitations characterize this study. First, we derived PM pollutant concentrations from big data to estimate individual exposures, potentially introducing exposure miscalculation. Additionally, factors like personal social information, capable of influencing outcomes, were omitted. The absence of ozone and nitrogen dioxide levels in our analysis poses another limitation, as their potential confounding effects remain unaddressed. Second, our study did not control for potential collinearity issues in the multiple models, possibly affecting the stability of the results. Additionally, using lag 01 to assess association between PM and CVD mortality may not capture prolonged exposure effects. Third, this study focused on PM_10_ and PM_2.5_, neglecting other pollutants that could contribute to cardiovascular mortality, thus limiting the comprehensiveness of our findings. Despite these limitations, our study contributes valuable insights into the association between PM pollutants and CVD mortality, emphasizing the need for further research to address these constraints and enhance the accuracy and applicability of future findings.

## 5 Conclusions

Our findings indicated significant associations between PM pollutants (PM_10_ and PM_2.5_) and CVD mortality in Shanghai, China. Moreover, our findings indicated that the health impacts of PM pollution may be varied by both seasonal and sociodemographic factors. These findings provide valuable insights that could guide the development of targeted policies at the intersection of environmental and social domains, thereby contributing to improved public health outcomes in the region.

## Data availability statement

The raw data supporting the conclusions of this article will be made available by the authors, without undue reservation.

## Author contributions

BF: Writing – original draft, Writing – review & editing. JW: Conceptualization, Data curation, Supervision, Validation, Writing – review & editing. LC (3rd author): Investigation, Methodology, Writing – review & editing. SJ: Investigation, Methodology, Writing – review & editing. QL: Investigation, Methodology, Writing – review & editing. RC: Investigation, Methodology, Writing – review & editing. NQ: Investigation, Methodology, Writing – review & editing. ZG: Investigation, Methodology, Writing – review & editing. LC (9th author): Investigation, Writing – review & editing. RS: Investigation, Methodology, Writing – review & editing. CW: Conceptualization, Data curation, Supervision, Writing – review & editing. WS: Conceptualization, Project administration, Supervision, Writing – review & editing.
